# Diplopia, COVID-19 and Vaccination: Results from a Cross-Sectional Study in Croatia

**DOI:** 10.3390/vaccines10091558

**Published:** 2022-09-19

**Authors:** Jelena Škunca Herman, Goran Marić, Maja Malenica Ravlić, Lana Knežević, Ivan Jerković, Ena Sušić, Vedrana Marić, Ivanka Petric Vicković, Zoran Vatavuk, Ozren Polašek

**Affiliations:** 1 Department of Ophthalmology, University Hospital Centre Sestre Milosrdnice, 10000 Zagreb, Croatia; 2 Croatian Institute of Public Health, 10000 Zagreb, Croatia; 3 Department of Public Health, University of Split School of Medicine, 21000 Split, Croatia; 4 Algebra LAB, Algebra University College, 10000 Zagreb, Croatia

**Keywords:** COVID-19, diplopia, vaccine, risk

## Abstract

The aim of this study was to explore diplopia as a symptom of undetected COVID-19 infection or as a possible side effect of COVID-19 vaccination. We examined 380 patients with diplopia admitted to the Department of Ophthalmology of the University Hospital Centre Sestre milosrdnice in Zagreb, Croatia, from July 2020 to June 2022. After excluding patients with confirmed organic underlying diplopia causes or monocular diplopia, we linked the patient information with the national COVID-19 and vaccination registries. Among the 91 patients included in this study, previously undetected COVID-19 infection as the possible cause of diplopia was confirmed in five of them (5.5%). An additional nine patients (9.9%) were vaccinated within one month from the onset of their symptoms, while the remaining 77 had neither and were therefore considered as controls. The breakdown according to the mechanism of diplopia showed no substantial difference between the vaccinated patients and the controls. We detected marginally insignificant excess abducens nerve affection in the COVID-positive group compared with that in the controls (*p* = 0.051). Post-vaccination diplopia was equally common in patients who received vector-based or RNA-based vaccines (21.4 vs. 16.7%; *p* = 0.694). COVID-19 testing should be performed for all cases of otherwise unexplained diplopia. The risk of post-vaccination diplopia was similar in both types of vaccines administered, suggesting a lack of evidence linking specific vaccine types to diplopia.

## 1. Introduction

Diplopia is an early sign of a muscular or neurologic pathological process affecting the eyes [1]. It reflects dysfunction of the extraocular muscles due to mechanical problems, neuromuscular junction disorders, cranial nerve palsy, supranuclear oculomotor pathway disorders, or toxin ingestion [2]. Diplopia can occur in various conditions, including strabismus, hypertension, diabetes, head and eye trauma, demyelination, malignancy, inflammation, infection, and autoimmune disease [3]. The most common causes of diplopia among autoimmune diseases include Graves’ disease, Hashimoto’s thyroiditis, Guillain–Barre syndrome, myasthenia gravis (MG), dermatomyositis, and systemic sclerosis [4]. Although diplopia occurs most often in Graves’ disease [5], myasthenia gravis was the leading cause of binocular diplopia-related ambulatory setting visits in the United States, according to De Lotts’ cross-sectional, population-based survey data [6]. Diplopia has been implied as a pathological sign associated with various infections [7], including acute viral infections [8,9,10,11,12]. Interestingly, it has also been reported as a possible anti-viral vaccination side effect [13] for polio [14], yellow fever [15], hepatitis B [16], or Japanese encephalitis [17].

The link between COVID-19 and diplopia has been a matter of ongoing debate, with various neuro-ophthalmologic symptoms and complications [18,19,20,21,22,23,24,25,26,27]. Interestingly, diplopia was also reported in asymptomatic SARS-CoV-2 infection, suggesting that it might also be a symptom of a previously undetected infection [28]. Several mechanisms causing diplopia were implied in COVID-19, including oculomotor nerve palsy [29,30,31], trochlear [32], or abducens nerve palsy [33,34,35,36,37,38,39,40,41]. The most likely underlying pathogenetic process involves immune reactions that are mediated by several classes of antibodies, namely anti-GQ1b immunoglobulin G (IgG) [42,43,44], previously being implicated in several neuro-ophthalmic disorders [45]; anti-GT1a IgG [44], involved in neurological disorders such as Guillain–Barre syndrome [46]; MOG antibodies [47], implicated in several myelin-related disorders [48]; or anti-GD1b, involved in neuromuscular junction disorders and MG [49]. Summarised evidence suggests that SARS-CoV-2 infection can trigger numerous autoimmune disorders [49], leading to unfavourable clinical outcomes [50]. In addition to being reported after acute SARS-CoV-2 infection, diplopia has also been reported by several previous studies after COVID-19 vaccination, raising concerns about this being possibly one of the less documented vaccination side effect [51,52,53,54]. The pathogenesis underlying these associations is unclear, but some interesting patterns can be identified. There is a substantial co-occurrence of COVID and MG, and both COVID infection and vaccines were implicated as a possible MG trigger [55]. Numerous underlying mechanisms were implicated, including molecular mimicry, immunosomes, and reactions related to “adjuvants” in vaccines [56,57]. A recent analysis reported an increased rate of vaccination side effects in a cohort of MG patients, with nearly 50% mortality in the unvaccinated subgroup of MG patients [57].

The COVID-19 pandemic has caused numerous disruptions in regular healthcare functioning, especially monitoring and surveillance [58]. Despite a quick response in Croatia and a functional data collection system deployed within weeks of the pandemic’s start [59], substantial changes and challenges in the diagnostics were identified [60] alongside constantly changing epidemic patterns, preventing the proper calculation of epidemiological rates [61,62,63].

Therefore, the aim of this study was to explore if incidental diplopia cases were associated with undetected COVID-19 or as a possible vaccination side effect based on the data from a tertiary-level clinical hospital in Croatia.

## 2. Materials and Methods

For the purposes of this study, we examined the medical records of all patients hospitalised for diplopia from 1 July 2020 to 1 June 2022 at the Department of Ophthalmology of the University Hospital Centre Sestre milosrdnice in Zagreb, Croatia. We included patients who were initially admitted with diplopia, including all who were assigned the following International Classification of Diseases (ICD-10) codes: H53.2 Diplopia; H49.0 Third (oculomotor) nerve palsy; H49.1 Fourth (trochlear) nerve palsy; H49.2 Sixth (abducent) nerve palsy; H49.3 Total (external) ophthalmoplegia; H49.4 Progressive external ophthalmoplegia; H49.8 Other paralytic strabismus; H49.9 Paralytic strabismus, unspecified; H51.0 Palsy of conjugate gaze; and H51.2 Internuclear ophthalmoplegia. Next, we performed a detailed search of their medical records, removing all cases of monocular diplopia. Additionally, we excluded all patients in which we could confirm restrictive strabismus (distyroid orbitopathy and extraocular muscle trauma), latent (heterophoria) and manifest strabismus of non-paralytic aetiology, tumour process of the central nervous system (CNS), and postoperative deficit involving the CNS, aneurysms and other causes (incomplete medical records, pansinusitis, and general endotracheal anaesthesia). Following this, we linked their data with the national COVID-19 and vaccination registries run by the Croatian Institute of Public Health to verify their infection and vaccination statuses. Based on this data linking, we classified all patients into three groups: likely COVID-19-caused diplopia (CoVd), likely post-vaccine diplopia (pVd), and controls (cc). In order to be classified as the likely COVID-19-caused diplopia, a patient had to be diagnosed with COVID-19 within one month of the diplopia onset (0–30 days). The second group, likely post-vaccine diplopia, was ascertained in those instances when a vaccine (the first or the second dose) was administered within one month from diplopia onset. Therefore, the onset of their diplopia had to occur within 30 days from vaccination. Lastly, patients diagnosed with diplopia who were not diagnosed with COVID-19 or were not vaccinated within the past month were considered a control group, reflecting the baseline risk of diplopia.

In all instances, diplopia was diagnosed by a detailed strabological clinical examination, aiming to identify paretic muscles. The examination included an eye movement examination in each of the nine gaze directions, a red glass test, a Bagolini striated glasses test (BSGT), and the measurement of the angle of ocular deviation (squint angle) at far and near distances using the alternate prism cover test (APCT).

### 2.1. Ethics

The study was based exclusively on secondary data, and no contact with the patients was made for the purposes of this study. The approval for this study was issued by the Ethical Panel of the University Hospital Centre Sestre milosrdnice, designated as document 251-29-11-21-01-2.

### 2.2. Statistics

Due to a small number of cases in two of the three groups analysed, the categorical data were analysed with Fisher’s exact test (two-tailed). The numerical data were analysed with a t-test. Two models of logistic regression were made, used to provide adjustment for the most apparent confounders. The model for likely post-vaccination diplopia was based on sex; age; presence of comorbidities (including diagnosed hypertension, hyperlipidaemia, diabetes mellitus, atrial fibrillation, cardiomyopathy, renal insufficiency, rheumatoid arthritis, and previous thrombotic events); and vaccine type, classified as vector-based vs. RNA-based. The vector-based vaccine type administered in Croatia is ChAdOx1, and the two RNA-based vaccines used in Croatia are BNT162b2 and mRNA-1273. The model for likely post-COVID diplopia included only sex, age, and the presence of comorbidities. In both cases, we assessed model fit using the Hosmer–Lemeshow test and correctly classified the number of cases from the classification table. All analyses were performed in R (www.r-project.org, accessed on 15 June 2022), with significance set at *p* < 0.05.

## 3. Results

There were 91 patients with binocular diplopia involved in this study, belonging to three study groups: likely post-vaccination diplopia (pVd; *n* = 9), diplopia in COVID-19 patients (CoVd, *n* = 5), and diplopia in patients who were neither confirmed cases nor vaccinated and were therefore considered as controls (cc; *n* = 77). The initial comparison of these groups revealed no significant differences in age, gender, and comorbidities existence, although both groups of cases were, on average, younger (Table 1).

The vaccine type was not associated with diplopia diagnosed within one month after vaccination vs. at any other time (Table 2).

Finally, we calculated the multivariate models, adjusted for the effects of age, sex, and comorbidities; notably, the post-vaccination model also included vaccine type (Table 3). Despite the very small number of cases, both models satisfied the diagnostic criteria (Hosmer–Lemeshow test *p* = 0.845 with 84.3% correctly classified cases for pVd, and *p* = 0.513 with 93.9% of correctly classified cases for CoVd). Notably, neither of the predictor variables reached statistical significance (Table 3).

## 4. Discussion

The results of this study might suggest that diplopia should be considered a possible symptom of a previously undetected COVID-19/SARS-CoV-2 infection. This is in line with the clinical appearance of the virus and previous reports, which showed a substantial tendency to affect cranial nerves and to cause neurological or ophthalmic symptoms [18,19,28,64]. Interestingly, we detected a nearly significant surplus of abducens nerve affections in COVID-19 cases, despite previous suggestions of the abducens nerve being the least commonly affected cranial nerve [26]. The difference probably arises from the sample composition; while the previous study focused on COVID-19-positive patients, this study was based on patients with diplopia. Nevertheless, one of the most salient results of this study is the recommendation to include SARS-CoV-2 testing as a mandatory element of the diagnostic protocol in otherwise unexplained diplopia.

This study did not provide a clear answer to the question of whether diplopia should be considered one of the possible side effects of COVID-19 vaccination. This finding is in line with several recent studies [57,65,66,67]. In addition to the common problems arising from vaccination side-effect surveillance [68,69], we were further limited due to an inability to calculate proper rates during the pandemic [62], as well as the organisational issue related to hospitals merging and gravitational areas changing, therefore disabling a more detailed time-series and rates analysis. However, this study did show that the underlying mechanisms of diplopia were similar in the controls and the post-vaccination group, suggesting that the risk of post-vaccination diplopia resembles the risk at baseline (vaccination-free). Furthermore, we detected no difference in the diplopia occurrence in patients who received vector-based vs. RNA-based vaccines. Previous comparisons often reflected substantial differences in the side-effect profiles between such vaccines [70], suggesting that the observed profile is more likely to reflect the baseline risk of diplopia and is unrelated to vaccination. However, such a finding should be replicated in an independent cohort before we can provide a more reliable answer to diplopia being considered a COVID-19 vaccination side effect. Notably, several other studies that investigated the ophthalmic side effects of the COVID-19 vaccine did not report diplopia as a side effect, further contributing to the low likelihood of diplopia arising from COVID-19 vaccination [71,72,73,74,75].

The limitations of this study are numerous, with most due to previously mentioned difficulties in obtaining comparable data during the epidemic and the peri-epidemic periods, with a spectrum of differences in the type of health care delivered during such times, which prevents direct comparisons and the calculation of epidemiological rates. Therefore, we did not provide the rates of events in this study, having considered them inherently biased. Additionally, any side-effect reporting system is always methodologically challenging [69,76], with possible upwardly biased estimates during the pandemic due to very prevalent awareness and even anti-vaccination campaigns. This limits direct comparisons of the side-effect rates with the pre-pandemic estimates [77]. This study further suffers from overall small sample sizes, meaning that it was prone to type I errors and that all of these conclusions cannot be used for a definitive answer but can only contribute to the overall discussion. In light of this, further data collection and analyses in independent cohorts and populations are required [27,78]. Clinical examination was another possible source of bias, so we could not establish the clinical course timeline or ascertain the viral and immunological dynamics. In addition, the inclusion of the Hess–Lancaster test could have provided an additional assessment element, but it was not performed. One of the limitations is related to the group of patients with unknown diplopia pathogenesis. This group is possibly the most interesting for understanding the disease pathogenesis and, possibly, the early stages of various other diseases, notably myasthenia gravis. A possible solution would include longer follow-up periods for such patients, during which their clinical manifestations would become more apparent. However, the overall evidence was insufficient to suggest the causal relationship between vaccination and diplopia occurrence, while the link between asymptomatic infection and diplopia seems more likely.

## Figures and Tables

**Table 1:** The comparison of the three study groups.

	Group			Significance (P)	
	pVd (Post-Vaccination)	CoVd (Undetected COVID)	cc (Controls)	pV vs. cc	CoV vs. cc
Age (years); mean ± SD	54.33 ± 18.70	52.40 ± 16.40	62.81 ± 18.39	0.195	0.221
Women; n (%)	5 (55.6)	4 (80.0)	40 (51.9)	~1	0.366
Comorbidities present; n (%)	6 (66.7)	3 (60.0)	49 (63.6)	~1	~1
Diplopia mechanisms; n (%)					
Cranial nerve (CN) paresis *					
CN III, right	1 (11.1)	0	3 (3.9)		
CN III, left	1 (11.1)	0	11 (14.3)		
CN III, binocular	0	0	2 (2.6)		
Subtotal, CN III	2 (22.2)	0	16 (20.8)	~1	0.577
CN IV, right	0	0	1 (1.3)		
CN IV, left	0	1 (20.0)	3 (3.9)		
CN IV, binocular	0	0	0		
Subtotal, CN IV	0	1 (20.0)	4 (5.2)	~1	0.276
CN VI, right	1 (11.1)	3 (60.0)	6 (7.8)		
CN VI, left	0	1 (20.0)	14 (18.2)		
CN VI, binocular	1 (11.1)	0	5 (6.5)		
Subtotal, CN VI	2 (22.2)	4 (80.0)	25 (32.5)	0.713	0.051
Internuclear ophthalmoplegia	1 (11.1)	0	0		
Vertical strabismus	0	0	1 (1.3)		
Cranial nerve neuritis	0	0	1 (1.3)		
Unknown pathogenesis	4 (44.4)	0	30 (39.0)		
Subtotal, other	5 (55.5)	0	32 (41.6)	0.473	0.151
Total	9	5	77		

* Due to a very low number of cases, only the sub-total number per nerve was tested statistically.

**Table 2:** Post-vaccine diplopia risk in vector- and RNA-based vaccinated patients.

Vaccination Type	Diplopia Occurrence		P
	Within One Month after Vaccination (*n* = 9)	At Some Other Time (*n* = 41)	
Vector-based vaccine	3 (21.4)	11 (78.6)	0.694
RNA-based vaccine *	6 (16.7)	30 (83.3)	

* Including different combinations of RNA-based vaccines.

**Table 3:** A logistic regression model used to explore the differences between likely post-vaccination diplopia (pVd) and likely COVID-caused diplopia (CoVd).

	Likely Post-Vaccine Diplopia (pVd)		Likely Post-COVID Diplopia (CoVd)	
Predictor Variable	OR [95% CI]	*p*	OR [95% CI]	*p*
Age	0.94 [0.88–1.01]	0.056	0.96 [0.90–1.02]	0.155
Sex				
Men (Ref.)	1.00		1.00	
Women	1.22 [0.27–5.58]	0.801	4.17 [0.43–40.38]	0.218
Comorbidities				
Not present (Ref.)	1.00		1.00	
Present	5.82 [0.50–67.09]	0.158	2.58 [0.22–29.72]	0.447
Vaccine type				
Vector-based (Ref.)	1.00		n/a	
RNA-based	0.43 [0.08–2.47]	0.347	n/a	

## Data Availability

The raw data are available upon reasonable request to the corresponding author.
